# Mandala painting therapy applied to cancer patient: a scoping review

**DOI:** 10.1007/s00520-025-09884-x

**Published:** 2025-09-24

**Authors:** Yue Chen, Yingping Fu, Xin Guo, Xiaoyan Mu, Yinglan Duan, Juli Shen

**Affiliations:** 1 Yunnan University of Traditional Chinese Medicine, Kunming, Yunnan Province China; 2 The Third People’s Hospital Of Yunnan Province, Kunming, Yunnan Province China

**Keywords:** Psychological care, Mandala painting therapy, Cancer patients, Scope review

## Abstract

**Purpose:**

Psychological care has been shown to effectively enhance the quality of life for cancer patients and reduce negative emotions such as anxiety and depression. Recently, mandala drawing therapy has emerged as a novel approach to assist cancer patients in managing their psychological challenges. Nevertheless, the application of this therapy lacks scientific guidelines, and inconsistencies persist across related studies. Therefore, this study aims to synthesize and scrutinize the existing research on the utilization of mandala drawing therapy for cancer patients, striving to establish a scientific framework for its widespread clinical implementation in the future and serving as a valuable resource for further research.

**Methods:**

A scoping review was conducted to delve deeper into the existing evidence and identify knowledge gaps relevant to future research efforts. Utilizing the Joanna Briggs Institute's methodology for systematic reviews, we conducted searches in PubMed, Web of Science, Cochrane Library, CINAHL, Embase, Wanfang, CNKI, VIP Information Services Platform, and China Biomedical Literature Service System, covering the entire duration from the establishment of each database up to December 2023.

**Results:**

The search strategy yielded 335 studies, of which 11 met the study criteria after screening. The findings indicated that mandala painting therapy effectively improved the quality of life and satisfaction with care among cancer patients while alleviating negative emotions. However, the treatment did not significantly affect pain levels and the fear of recurrence in these patients.

**Conclusion:**

Mandala painting therapy primarily functions as a psychotherapeutic and psychological assessment tool. Although it has shown positive effects on cancer patients, further exploration is needed regarding its implementation methods. Future research should consider expanding the sample size and making more substantial efforts to investigate the therapy's effects in depth.

**Trial registration:**

OSF registration DOI: https://doi.org/10.17605/OSF.IO/EZNMX

**Supplementary Information:**

The online version contains supplementary material available at 10.1007/s00520-025-09884-x.

## Novelty Statement

### Step 1: Identifying the research questions

1. Relevance Statement

Mandala painting therapy is not restricted by language, culture, age, location, or disease, enjoys high patient acceptance, and is simple to implement and operate, making it widely applicable in clinical practice.

Mandala painting therapy can help alleviate negative emotions in cancer patients, such as anxiety and depression, improve treatment compliance, and thus significantly improve patients' quality of life.

2. What this paper adds

Few studies have investigated whether there are differences in the application of various intervention protocols for Mandala Painting Therapy in cancer patients and whether there are discrepancies in the beneficial effects observed across different studies.

To better implement Mandala Painting Therapy in the future, there is a need to further understand the similarities and differences between studies and to explore the most standardized protocol for implementing Mandala Painting Therapy.

## Introduction

Cancer is the second leading cause of death globally, responsible for nearly one in six deaths and three in ten premature deaths (ages 30–69) worldwide [1]. In January 2024, the American Cancer Society (ACS) published the 2024 U.S. Cancer Statistics Report, indicating that the number of new cancer cases in the United States surpassed 2 million for the first time [2]. The Global Cancer Statistics Report 2022, released in April 2024, estimates that there are approximately 19.976 million new cases of cancer worldwide. The number of new cancer cases in China is estimated to be about 4.82 million [3]. The incidence of cancers and resultant mortality is increasing worldwide, imposing a significant physical, emotional, and economic toll on individuals, families, communities, and healthcare systems [4, 5]. In the diagnosis and treatment of cancer patients, the prolonged duration of the disease and complex treatment regimens often cause significant physical and mental pain. Common treatment-related adverse effects include fatigue, hair loss, appetite loss, constipation or diarrhea, sleep problems, pain, nausea or vomiting, and more [6]. Common psychological issues include pain, anxiety, depression, and elevated stress as a result of the significant financial costs associated with the illness and its care [7]. This psychological distress can lead to various problems, affecting not only treatment adherence but also reducing the patients' overall quality of life. Traditional individual psychological interventions may be inadequate to address these complexities, thus necessitating the exploration of innovative technological advancements in the psychological domain.

One notable innovation is the utilization of mandalas as both a therapeutic and evaluative tool. Carl Jung, a distinguished psychologist, was the first to propose the therapeutic potential of mandala drawing. He posited that creating a mandala induces a calming and healing effect on the individual, promoting spiritual integration and fostering a deeper sense of meaning in life [8]. Mandalas are also recognized as a creative outlet for revealing trauma, symbolically organizing and integrating emotions and experiences, akin to narrative writing [9]. Through shapes and colors, painters can express their inner thoughts, while mandala paintings reveal unconscious conflicts symbolically. Leveraging the unique integrative function of mandalas, this process resolves internal contradictions, achieving harmony and stability. Grounded in Jung's psychoanalytic theory of self-nature, mandala painting possesses a robust theoretical foundation. Additionally, empirical studies indicate that mandala painting not only serves as an evaluative tool but also exhibits therapeutic efficacy. Consequently, mandala painting represents a mature psychological assessment and treatment technique worthy of promotion, particularly in cancer-related mental health services. However, the intervention protocols, evaluation metrics, and therapeutic outcomes of mandala drawing therapy in cancer patients remain inconsistent. Substantial heterogeneity persists across related research domains, lacking a standardized scientific basis for nursing workflows. A scoping review, an evidence-based research methodology, can systematically explore the scope and breadth of a research area, synthesize and disseminate findings, and identify gaps and deficiencies in existing research. This approach is especially suited for complex or under-explored fields, clarifying research problems and directions [10]. Adhering to Arksey et al.'s methodological framework for scoping reviews [11], this study summarizes and analyzes research on mandala painting therapy in cancer patients, aiming to provide valuable insights for healthcare professionals pursuing further investigation.

## Methods

This review was conducted using the Joanna Briggs Institute methodology for scoping reviews [12, 13] and reported by the Preferred Reporting Items for Systematic Reviews and Meta-Analyses extension for Scoping Reviews (PRISMA-ScR) guidelines [14]. The framework includes six steps:

1. Identifying the research questions;
2. identifying relevant literature;
3. study selection;
4. charting the data;
5. summarizing and analyzing the data;
6. reporting the results.

## Protocol and registration

Before conducting this scoping review, we clearly defined the objectives, inclusion criteria, and a comprehensive methodology. Additionally, the review protocol has been officially registered on the Open Science Framework (OSF), with a registration DOI available at 10.17605/OSF.IO/EZNMX.

## Search strategy and information sources

### Step 2: Identification of relevant literature

The search strategy followed a three-step methodology to identify both published and unpublished studies. Initially, a preliminary search was conducted in PubMed and CNKI to identify keywords, which allowed for an analysis of text words present in the titles, abstracts, and index terms. Subsequently, the search strategy was refined and tailored for each additional database. Finally, the reference lists and citations from the ultimately selected studies were screened to identify any additional eligible studies. The search will be conducted from its inception until December 2023, with the results limited to Chinese and English language articles.

## Process of selecting sources

### Step 3: Study selection

Two researchers independently conducted the literature screening and data extraction process, with team members assisting in resolving any disagreements. The specific methods involved searching according to the established search strategy, excluding retrieved literature, importing it into NoteExpress software to eliminate duplicates, and then conducting both initial and re-screening based on the NERF criteria. This process was followed by cross-checking to finalize the included literature and performing a summary analysis.

The extracted data encompassed several categories:

(i) authorship, study type, country and year of publication, research object, sample size, and cancer treatment stage;
(ii) intervention methods employed in the experimental group, including the tools used, researchers involved, type of intervention content, frequency and duration of the intervention, as well as the implementation setting;
(iii) intervention methods utilized in the control group; and
(iv) endpoint indicators and their respective measurement tools.

## Eligibility criteria

In selecting studies for this review, we used broadly defined inclusion criteria that considered population, context, and concept, which is a common practice in scoping reviews [13, 14]. The research team created an inclusion and exclusion criteria table to ensure consistent selection of studies (see Table 1).

**Table 1:** Inclusion and exclusion criteria for study selection

Inclusion	Exclusion
The trial included individuals diagnosed with cancer The research topic involves the application and evaluation of mandala painting therapy's impact on cancer patients, encompassing aspects such as mandala painting therapy, mandala painting, and mandala therapy The literature review includes primary research such as randomized controlled trials, non-randomized controlled trials, case–control studies, cohort studies, case studies, qualitative studies, and so on The language used is either Chinese or English	Literature on Mandala Painting Therapy, when combined with other interventions, demonstrates the effects of Mandala Painting. However, there is a lack of effective measurement for therapy outcomes The literature available is incomplete or ambiguous, with full texts often being unavailable Studies found in published literature have been replicated Non-peer-reviewed conference papers and posters are also included

## Selection of evidence

During the review process, the research team raised concerns about the use of painting therapy for cancer patients. The scoping review was specifically designed to gather relevant information on mandala painting therapy for these patients, rather than to provide evidence for its practical application. As a result, it was essential to restrict the application of mandala painting therapy solely to cancer patients. Consequently, the research team decided to exclude articles that combined mandala painting therapy with other interventions, ensuring that the findings were exclusively relevant to mandala painting therapy.

## Data extraction and synthesis

### Step 4: Data abstraction

Two independent reviewers, the second and third authors, carefully extracted relevant data from each of the included articles. This data was then reviewed by various team members, who resolved any uncertainties or ambiguities through thorough discussions. Following our established protocol, we compiled the extracted data into a tabular format to address our research questions. The summarized elements included general characteristics of each study, such as the authors, year of publication, study type, country, study population, timing of the intervention, duration of the intervention, and form of the intervention. Additionally, we incorporated specific findings related to mandala painting therapy into the summary.

## Results

### Selection of sources of evidence

#### Step 5: Data synthesis

**Fig. 1:**
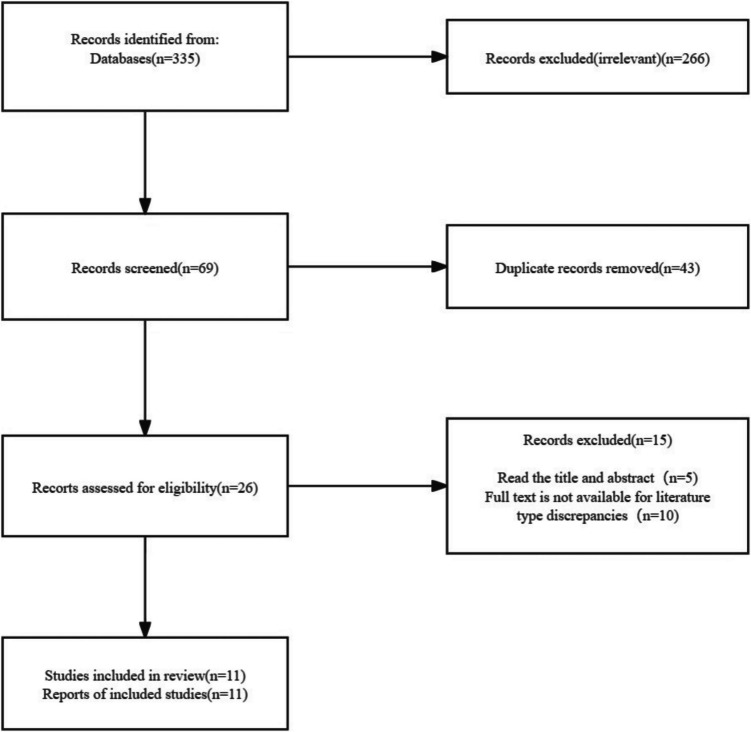
Flowchart depicting the study selection process

After conducting a preliminary search, we retrieved a total of 335 pieces of literature. From these, we excluded 266 articles due to their inconsistency with the research topic and objectives. Additionally, we removed 43 duplicate articles, which left us with 26 remaining articles. Upon reviewing the titles and abstracts, we further excluded 5 articles. We then read the full texts of the remaining 21 articles, excluding an additional 10 that did not meet the required criteria or were unavailable in full text. Ultimately, 11 studies that satisfied our inclusion criteria were selected for analysis, as shown in Fig. 1 and Table 2.

**Table 2:** Characteristics of included studies and their frequencies

	n (%)	Included studies
Publication year		
2000–2010	9%	Elkis-Abuhoff D et al.(2009)
2011–2022	73%	Yaoli Li et al.(2017),Shufen Zhao et al.(2017),Shufen Zhao et al.(2019),Gürcan M and Atay Turan S(2020),Gürcan M and Atay Turan S(2021),Yakar H K et al.(2021),Lili Sun et al.(2021),Lingyu Su et al.(2022)
2023	18%	Moharamkhani M et al.(2023),Akbulak F and Can G(2023)
Research design		
Qualitative studies	18%	Elkis-Abuhoff D et al.(2009),Gürcan M and Atay Turan S(2020)
Quantitative studies		
Controlled trial	55%	Yaoli Li et al.(2017),Shufen Zhao et al.(2017),Shufen Zhao et al.(2019),Gürcan M and Atay Turan S(2020),Gürcan M and Atay Turan S(2021),Lili Sun et al.(2021),Lingyu Su et al.(2022)
Type of experimental studies	27%	Yakar H K et al.(2021),Moharamkhani M et al.(2023),Akbulak F and Can G(2023)
Country of origin		
China	46%	Yaoli Li et al.(2017),Shufen Zhao et al.(2017),Shufen Zhao et al.(2019),Lili Sun et al.(2021),Lingyu Su et al.(2022)
Turkey	36%	Gürcan M and Atay Turan S(2020),Gürcan M and Atay Turan S(2021),Yakar H K et al.(2021),Akbulak F and Can G(2023)
United States Of America	9%	Elkis-Abuhoff D et al.(2009)
Iran	9%	Moharamkhani M et al.(2023)

## Characteristics of sources of evidence

Table 2 provides a summary of the key characteristics of the studies included in this review. Most of the studies employed quantitative methods (*n* = 9), which consisted of controlled trials (*n* = 6) and experimental studies (*n* = 3). Additionally, the review included two qualitative studies. Of the studies reviewed, ten were published between 2017 and 2023, with the majority conducted in China (*n* = 5). The primary focus of the inclusion criteria was on the psychotherapeutic application of mandala drawing therapy. Among the qualitative studies, one utilized mandala drawings to explore the experiences of hospitalized adolescents undergoing cancer treatment, while the other aimed to use mandala drawing therapy as a psychological assessment tool for cancer patients. The studies involved patient populations with at least one broad cancer diagnosis. Detailed information regarding the treatment duration, the researcher conducting the study, the timing and length of the intervention, as well as the form of the intervention and outcome measures for the study participants, is provided in Table 3.

**Table 3:** General characteristics of the included studies

Inclusion of literature	Published (year)	Country or region	Research object	Research design	Research objective	Interventionists	Time of intervention /Duration	Forms of intervention
Yakar H K et al.[15]	2021	Istanbul	Cancer patient	Type of experimental re- search	Period of inactive treatment	Trainers, researchers who have participated in training	One day per week for 8 weeks,2h/session	Unstructured mandalas accompanied by Far Eastern and classical music in languages other than the individual's native language
Lili Sun et al.[16]	2021	China	Cancer patient	A ra- ndomised controlled trial	Period of chemotherapy	Teams that have participated in training,including counsellors, etc	2 cycles of chemotherapy per cycle for a total of 10 cycles	Structured mandala with calming music
LingYu Su et al.[17]	2022	China	Patients with primary liver cancer	A ra- ndomised controlled trial	Operative period	Teams that have participated in training,including counsellors,nurses,graduate stud- ents	1 intervention per day for a total of 6 sessions of 50-60min duration	Structured mandala with soothing soft music
YaoLi Li et al.[18]	2017	China	Terminal cancer patient	A ra- ndomised controlled trial	End-stage cancer	nurses	Once a week, give enough time	Not mentioned, soothing soft music plus meditation
ShuFen Zhao et al.[19]	2017	China	Non-elderly cancer patients	A ra- ndomised controlled trial	Treatment period	Psychological counsellors, trained members of hospital psychological care teams, oncology nurses	2 times a week, 45 min each time, 15:00 ~ 15:45	Structural Mandala, no
Gürcan M et al.[20]	2021	Istanbul	Adolescent cancer patients	A ra- ndomised controlled trial	End-stage cancer	Fellows who have attended mandala courses	Two times in total, at intervals of 2 or 3 days, with time taken into account for adolescents' recommendations	Unstructured mandala accompanied by classical or instrumental music
ShuFen Zhao et al.[21]	2019	China	Patients with esophageal cancer	A ra- ndomised controlled trial	Radiation therapy period	Members of hospital care psychology teams, psycholog -ists who have participated in the training	1 time/week, 6 weeks in total, 40min/time, 15:30 ~ 16:10	Structural Mandala, no
Akbulak F et al.[22]	2023	Istanbul	Female breast cancer patients	Type of experimental research	Period of chemotherapy	Nurses	Total 1 time, 30min	Structural Mandala, no
Moharamkhani M et al.[23]	2023	Iran	Childhood cancer patients	Type of experimental research	Not in advanced stages	Researcher	Daily, in 6 sessions for 6 days, 45 min each session (11:00 a.m.)	Structural Mandala, no
Gürcan M et al.[24]	2020	Istanbul	Adolescent cancer patients	Qualitative research	Treatment period	Researcher	Total 1 session, 1–2 h	Unstructured mandala, accom -panied by classical or instrumental music
Elkis-Abuhoff et al.[25]	2009	United States of America	Female breast cancer patients	Qualitative research	Treatment period	Doctors	Every time you go to the doctor	Unstructured mandala, no

## Results of individual sources and synthesis of data extracted

We conducted a thorough analysis of the different intervention protocols used in the trials and the outcomes they produced. Our findings have been systematically compiled and are presented in Table 3. From this synthesis of protocols, we made three main observations:

- there is a lack of standardization in intervention protocols;
- there is ambiguity surrounding the delivery method and benefits of Mandala painting therapy;
- there is an insufficient analytical evaluation of Mandala painting therapy.

## Ways of doing mandala painting therapy

In clinical settings, mandala painting is utilized in two primary forms: unstructured and structured [26]. Structured mandalas offer individuals a pre-designed template for drawing, thereby eliminating the need for self-design. In contrast, unstructured mandalas involve drawing within a defined circle without the aid of a template [27]. Carl Jung advocated for the unstructured approach. Among the articles reviewed in this study, four utilized unstructured mandalas, while seven employed the structured format. Notably, five articles from China favored structured mandalas, which are rooted in mandala painting psychotherapy theory or use templates created by experts such as Chen CR. The remaining articles did not clarify the reasoning behind their choice, leading to a lack of transparency regarding the principles guiding clinical selection. Most studies that incorporated mandala painting also integrated musical composition. Yakar H.K. et al. outlined a program that combined weekly mandala creation with various meditation techniques, typically accompanied by music, except during silent meditation sessions [15].

## Intervention programme in mandala painting therapy

The development stage and personnel involved in mandala painting therapy span various phases of cancer treatment, including the pre-treatment period [15], chemotherapy period [16, 22], operative period [17], and radiation therapy period [21]. Some articles, however, do not specify the treatment period [18–20, 23–25], suggesting that this therapy is not consistently applied throughout the entire treatment process. Mandala painting therapy is facilitated by individuals or teams responsible for developing and executing the intervention program. These practitioners are typically nurses, researchers, or the authors themselves, while teams may also include counselors, nurses, postgraduate students, or psychological trainers. The composition of these practitioners is crucial for ensuring the therapy's effectiveness and accuracy. The intervention programs are implemented through group training and individual training sessions, with the number of participants in group training varying. For instance, Sun LL et al. [16] controlled the group size to fewer than three individuals, while Li YL et al. [18] involved all patients in the intervention, and Zhao SF et al. [19] divided patients into four separate groups. Another study reported having 6–8 individuals per group[21].

The frequency and duration of mandala drawing therapy varied significantly among the 11 studies included in the review, revealing a lack of standardized implementation protocols. The therapy duration correlated with the entire intervention period, ranging from a single day to five chemotherapy cycles. The number of interventions varied from one to ten, with the duration of a single session lasting between 30 min and an unspecified length. Notably, most Chinese studies conducted the intervention in the afternoon, primarily after 3:00 PM.

In total, eleven articles addressed the intervention schedule for mandala painting therapy. Three articles recommended one intervention per week, two required daily interventions, one specified two interventions per week, another mandated two interventions per chemotherapy cycle, and the final article called for one intervention per treatment. Among the articles, seven reported intervention times lasting less than one hour, two indicated durations of one to two hours, one did not specify the duration, and another reported unlimited time lengths.

Ten articles explored the settings in which mandala painting therapy is conducted. However, there is currently no standardization for the environments in which this therapy is delivered. Many previous studies did not limit the delivery settings, resulting in varying conditions that have not produced a clear and consistent impact on the effectiveness of mandala drawing therapy.

## Analysis and evaluation of mandala painting therapy

The analysis and evaluation of mandala paintings vary based on two distinct forms: unstructured and structured mandalas. Unstructured mandalas involve three main steps: naming the artwork, listing the colors used, and identifying the numbers and figures present in the piece. In contrast, structured mandalas primarily focus on analyzing the themes and colors within the artwork.

Regarding the evaluation of mandala drawing therapy, only one of the included studies [14], specifically targeting cancer patients, utilized a questionnaire to assess six aspects of the implementation of mandala drawing therapy. These aspects include the frequency of sessions, content, environment, duration of the intervention, length of each session, and overall satisfaction with the intervention activities.The overall patient satisfaction rate was 98.3%. To evaluate the effectiveness of mandala painting therapy for cancer patients, the quantitative studies included in the assessment reported various psychological indicators, primarily measured through questionnaires. The main categories of these indicators included anxiety, depression, mental toughness, and overall distress/mood.

Additionally, three trials reported physical indicators, such as sleep quality (assessed using the Pittsburgh Sleep Quality Index), illness confidence, and quality of life (measured by the Quality of Life Rating Scale and the Quality of Life Scale). Two trials also examined other indicators, including patient satisfaction with nursing care and satisfaction with mandala drawing therapy.

The two qualitative studies were assessed primarily by the researchers. Table 4 provides further details. Most trials reported positive effects of mandala painting therapy on both physical and psychological indicators among cancer patients. However, some trials found no significant impact of the therapy on patients' distress or fear of recurrence. Notably, one study suggested that mandala drawing therapy could be beneficial for patients with high levels of anxiety before chemotherapy.

**Table 4:** Outcome indicator of the included studies

Inclusion of literature	Psychological indicators	Other indicators
Yakar H K et al.[15]	Distress thermometer State/Trait Anxiety Inventory STAI	/
Lili Sun et al.[16]	positive And negative affect scale,PANAS	/
LingYu Su et al.[17]	self-rating anxiety scale,SAS self-rating depression scale,SDS	pittsburgh sleep quality index,PSQI Mandala painting therapy satisfaction questionnaire
YaoLi Li et al.[18]	self-rating anxiety scale,SAS self-rating depression scale,SDS	Nursing satisfaction questionnaire Quality of life rating form
ShuFen Zhao et al.[19]	self-rating anxiety scale,SAS	/
Gürcan M et al.[20]	The Hospital Anxiety and Depression Scale,HADS The Memorial Symptom Assessment Scale,MSAS	/
ShuFen Zhao et al.[21]	self-rating depression scale,SDS	quality of life questionnaire,QLQ-C30
Akbulak F et al.[22]	The distress thermometer The State–Trait Anxiety Inventory	/
Moharamkhani M et al.[23]	Spielberger State-Trait Anxiety Inventory	/
Gürcan M et al.[24]	/	/
Elkis-Abuhoff et al.[25]	/	/

## Discussion

### Step 6: presentation of results

The main goal of this review is to explore the methods used for implementing mandala painting therapy, identify the challenges of existing intervention programs for cancer patients, and highlight the shortcomings in analytical evaluation. This will provide a foundation for future research that healthcare professionals can pursue.

### Nature of current evidence, gaps, and implications for research

#### Mandala painting therapy may assist cancer patients in alleviating negative emotions and enhancing their quality of life. However, the method of implementation and its specific benefits require further investigation

Mandalas, as an art therapy tool, come in two forms: structured mandalas and unstructured mandalas. Eight articles utilized structured mandalas, while three used unstructured mandalas. Cornell [28] mentioned that while coloring a mandala, or drawing a structured mandala, the patient becomes engrossed in the activity, forgetting daily routines, worries, and anxieties, which leads to a state of comfort, calmness, and focus on the coloring process. Thus, the level of attention involved in coloring determines the effectiveness of the art therapy tool. Curry et al. [29] also confirmed that structured mandala coloring helps reduce anxiety and that structured coloring is more effective. Curry also suggested that future research could test whether creating mandalas reduces anxiety. On the other hand, the study by Van der Vennet et al. [30] found that structured mandalas were not commonly used compared to creating original mandalas. The first experimental study to use mandalas as an intervention, Slegelis et al. [31], used unstructured mandalas. Structured mandalas are primarily colored on fixed templates, and their inherent structure may be somewhat restrictive, hindering the natural flow of mental energy. Zhou XG et al. [32] also suggested that a combination of structured and unstructured mandalas could allow subjects to express themselves creatively and mobilize the freedom of mental energy.In conclusion, structured and unstructured mandalas each have their own advantages and disadvantages, and a principle of choice needs to be developed to improve the accuracy and reliability of the experiment.

Regarding the positive effects of mandala interventions on anxiety, Carl Jung stated in his Mandala Symbolism that mandalas have a calming and healing effect on people experiencing trauma [8]. Forzini et al. [33] reported that painting during chemotherapy can make patients feel positive and express their emotions, concluding that art therapy may help reduce chemotherapy-related stress. The research conducted by Li YL et al. [18] revealed that mandala painting therapy has the potential to alleviate psychological and physical symptoms arising from cancer, thereby enhancing patients' adherence to treatment and ultimately improving their overall quality of life. All 11 included articles indicated that mandala painting therapy can alleviate negative emotions such as anxiety and depression and improve the quality of life in cancer patients.Similarly, a study led by J.C. Cohen et al. [34] underscored the beneficial impact of mandrake on depression and bodily symptoms among breast cancer patients. However, about the specific beneficial effects of mandala drawing therapy on anxiety in cancer patients, another result of the study by Moharamkhani M et al. [23] showed a statistically significant relationship between the number of hospitalizations and anxiety levels in children with cancer. Other studies have shown that children who have not been hospitalized before and those who have had fewer previous operations show more anxiety than those who are already familiar with the medical environment [35, 36]. Further research is required to confirm any potential relationship between the number of hospitalizations and anxiety levels. Geue et al. investigated the effect of art therapy on the quality of life of patients with higher and lower levels of distress and observed statistically similar changes in quality of life scores for the two groups, suggesting that it is not possible to determine which group of patients might benefit more from art therapy. A study by Akbulak F et al. [22] showed that mandala painting therapy may be a useful approach for patients with high levels of anxiety before chemotherapy and suggests that the relationship between pre-treatment levels of distress and art therapy outcomes needs to be explored in the future to determine which patient groups may benefit most from art therapy interventions.

#### Although Mandala painting therapy is not widely spread, there is a lack of uniformity in the development of intervention programs

Jung's mandala painting therapy has played a key role in its promotion and widespread adoption since 2017 [37]. In terms of its execution, Zhao SF et al. [19] attributed their choice of structured mandalas to significant differences in patients' cognitive levels. For example, Li YL et al. [18] demonstrated that mandala painting combined with music effectively improved patients' emotional states. In contrast, Chen CR et al.'s study emphasized that the drawing process itself facilitated a meditative state and the mobilization of positive emotions. A meta-analysis further revealed that relaxation therapies, including mindfulness and meditation, can enhance mental health and alleviate symptoms of anxiety and depression [38]. Xu ZJ [39] and others have supported the combined use of mandalas and meditation during the painting process, with participants often experiencing therapeutic benefits after meditating post-mandala creation. The study conducted by Su LY et al. [17] revealed that experts were consulted during the initial design phase of the intervention program to bolster its scientific validity. Furthermore, some articles mention that these practitioners undergo training in mandala painting therapy, potentially enhancing the therapy's delivery. Training in Mandala painting therapy before the study enhanced the professionalism of leaders and ensured the program's effectiveness.

Determining the most effective delivery phase for mandala drawing therapy and personnel remains a challenge. Su LY et al. [17] specifically chose the intervention time between 15:00 and 17:00 to avoid disrupting the patient's treatment and rest schedule. Gürcan M et al. [20] emphasized the importance of considering the patient's suggestions in selecting the appropriate intervention time. Regarding the intervention cycle, Su LY et al. [17] based their intervention cycle on the drawing therapy intervention cycle established by Li Q et al. [40]. The frequency of interventions was determined by considering the average number of hospital days for HCC patients undergoing RFA. Conversely, Altay et al. [41] observed that interventions lasting more than a week, specifically two mandala interventions, might reduce participants' interest. Gürcan M et al. [20] noted that adolescents often discharge within a week of completing their chemotherapy, suggesting that future studies could limit the number of interventions to 2 times a week. Various treatment periods can be explored to identify the optimal duration.Clear guidelines are lacking for personnel requirements, intervention format, duration, and timing, but studies by Su LY et al. [17] suggest a time frame of 15:00–17:00, with intervention frequency based on research by Altay et al. [41] and Gürcan M et al. [20] recommending up to 2 sessions per week. According to Akbulak F et al. [22], the majority of studies exploring the impacts of art therapy implemented the interventions in a hospital setting, specifically in designated areas for artistic activities. Gürcan M et al. [20] observed that adolescents' mental states or moods can undergo rapid changes in stressful and traumatic environments like hospitals. The environment implementation in two studies by Akbulak F [22] and Gürcan M [20] focused on hospital settings with designated areas for art therapy. Each study had unique intervention protocols. The Chinese study on mandala painting therapy for cancer patients, based on the theoretical framework of Chen CR et al. [26], utilized painting templates from Canrui Chen's [42] "Mirror of the Mind: Mandala Painting Therapy," aligned with mandala psychotherapy principles. The Turkish article drew heavily from Curry et al.'s [29] research to shape its program development. In the future, healthcare practitioners should explore the development of a specific form of intervention for the implementation of mandala painting therapy to ensure that the therapy can be maximized.

#### The analytical evaluation of mandala painting therapy requires improvement, and its long-term effects need further verification.

The information provided can be incorporated into the analysis and evaluation to enhance the effectiveness of the therapy, thereby not hindering freedom.

Our current investigation has revealed that the effectiveness of mandala drawing therapy may vary based on the age of the patients. For example, among adolescent cancer patients, this therapy significantly reduces anxiety and helps them overcome their fears, while also boosting their confidence in the fight against the disease. Breast cancer patients benefit from Mandala painting therapy, as it offers therapeutic relief and serves as a platform for expressing emotions during treatment. Post-operative liver cancer patients experience improvements in anxiety and depression levels, better sleep quality, and generally high patient satisfaction. For patients with advanced cancer, mandala painting therapy plays a crucial role in promoting psychological balance, enabling them to approach cancer with a positive mindset, enhancing the value of their lives, extending their lifespan, improving compliance with treatment, and fostering the confidence and courage needed to overcome the illness. However, for patients undergoing their first course of chemotherapy, while mandala painting therapy may show promise in reducing anxiety, further research is needed to conclusively determine its effectiveness in managing anxiety associated with this initial phase of treatment. Although mandala drawing therapy effectively alleviates anxiety and depression, it demonstrates limited impact on physiological markers like pain. Yakar H K et al. [15] argued that cancer patients often experience an increase in pain levels due to the psychological burden of anxiety, the physical consequences of social isolation, and insomnia, considering pain as a psychosocially and physically distressing experience. Furthermore, the research by Gürcan M et al. [24] highlighted the potential long-term impact of social isolation, extending into adulthood, and the need for interventions to mitigate loneliness. Therefore, future research could explore ways to reduce patients' anxiety, social isolation, and insomnia to enhance the therapeutic effectiveness.

A study conducted by Han et al. [43] implies that the depression and despair experienced by esophageal cancer patients also profoundly impact the psychological well-being of their companions. Consequently, it is crucial to provide psychological interventions to both the patient and their companion. Similarly, a study led by Akbulak F et al. [22] demonstrated that the presence of relatives during treatment can significantly alleviate patients' anxiety and enhance their relaxation levels. Therefore, when patients express a desire for their family members to participate, it is advisable to involve them in such studies to effectively reduce the patient's anxiety. Furthermore, Moharamkhani M et al. [23] reported that hospitalized children and their parents actively participated in the intervention session. In another study, Zhou XG et al. [32] applied the mandala painting technique to caregivers of cancer patients, resulting in improved physical and psychological well-being for these caregivers. Additional studies [44–46] have also emphasized the need for concurrent psychological interventions for cancer patients suffering from depression and anxiety, as well as their caregivers, to enhance the quality of life for both. Notably, Moharamkhani M et al. [23] also observed that oncology nurses may develop psychological issues over time, indicating that the application of mandala drawing therapy could potentially benefit them as well. Consequently, it is advisable to consider incorporating mandala drawing therapy into the care plans for both caregivers and oncology nurses in the future. This approach should prioritize the assessment of individual psychological states and needs, taking into account the unique circumstances of each individual [32, 47].

## Limitations

This review has several limitations. Firstly, it only included articles in Chinese and English, which could lead to publication bias. Secondly, the quality of the extracted articles was not evaluated, limiting the strength of the argument. Thirdly, mandala painting therapy has been less commonly practiced and lacks support from large sample randomized controlled trials.

## Conclusions

This scoping review discusses how mandala drawing therapy can effectively enhance negative emotions such as anxiety and depression in cancer patients and improve their quality of life. Currently, this therapy is not widely utilized among cancer patients and is in the early stages of research. In the future, we should explore a standardized intervention protocol for mandala drawing therapy that can benefit family caregivers and oncology nurses. This can be combined with other psychological care techniques or artificial intelligence technology to continuously enhance the intervention protocol's effectiveness. Additionally, more high-quality randomized controlled trials and follow-up observations are needed to validate the therapy's long-term application.

## Supplementary Information

Below is the link to the electronic supplementary material.

Supplementary file1 (DOCX 83 KB)

Supplementary file2 (DOCX 26 KB)

## Data Availability

No datasets were generated or analysed during the current study.
