# Supplementary material for: Mandala painting therapy applied to cancer patient: a scoping review
Source: Support Care Cancer. 2025 Sep 24;33(10):875. doi: 10.1007/s00520-025-09884-x (PMC12460571; doi:10.1007/s00520-025-09884-x)
Supplement: Supplementary file 1 — Supplementary file1 (DOCX 83 KB) [file 520_2025_9884_MOESM1_ESM.docx]

**Search terms and strategies**

Full search terms and strategies in **Chinese electronic databases** including CNKI, Wanfang Knowledge Service platform, VIP resource integration service platform, and China Biomedical Literature Database

#1 (Mandala Painting Therapy OR Mandala Painting OR Mandala OR Mandala Painting Treatment OR Mandala Therapy OR Mandala Treatment)

#2 (neoplasms OR neoplasm OR cancer OR tumor OR neoplasia OR malignant neoplasm* OR malignancy)

#1 AND #2

No restrictions were set on literature type or publication status.

**PubMed**

Search: (“Mandala Painting Therapy”［Ti/Ab］OR“Mandala Painting”［Ti/Ab］OR“Mandala”［Ti/Ab］OR“Mandala Painting Treatment”［Ti/Ab］OR“Mandala Therapy”［Ti/Ab］OR“Mandala Treatment”［Ti/Ab］) AND (“neoplasms”［Mesh］OR“neoplasm”［Ti/Ab］OR“cancer”［Ti/Ab］OR“tumor”［Ti/Ab］OR“neoplasia”［Ti/Ab］OR“malignant neoplasm*”［Ti/Ab］OR“malignancy”［Ti/Ab］)

**Web of Science**

You searched for : (TS= (Mandala Painting Therapy OR Mandala Painting OR Mandala OR Mandala Painting Treatment OR Mandala Therapy OR Mandala Treatment)) AND (TS= (neoplasms OR neoplasm OR cancer OR tumor OR neoplasia OR malignant neoplasm* OR malignancy))

**Embase**

1 Mandala Painting Therapy':ab,ti

2 Mandala Painting':ab,ti

3 Mandala':ab,ti

4 Mandala Painting Treatment':ab,ti

5 Mandala Therapy':ab,ti

6 Mandala Treatment':ab,ti

8 1 or 2 or 3 or 4 or 5 or 6

9 neoplasms':ab,ti

10 neoplasm':ab,ti

11 cancer':ab,ti

12 tumor':ab,ti

13 neoplasia':ab,ti

14 malignant neoplasm*':ab,ti

15 malignancy':ab,ti

16 9 or 10 or 11 or 12 or 13 or 14 or 15

17 8 and 16

**The Cochrane Library**

#1 Mesh descriptor: [Mandala Painting Therapy] explode all trees

#2 (Mandala Painting Therapy OR Mandala Painting OR Mandala OR Mandala Painting Treatment OR Mandala Therapy OR Mandala Treatment)

#3 #1 or #2

#4 Mesh descriptor: [cancer] explode all trees

#5 (neoplasms OR neoplasm OR cancer OR tumor OR neoplasia OR malignant neoplasm OR malignancy)

#6 #4 and #5

Table 1 Inclusion and exclusion criteria for study selection.

| Inclusion | Exclusion |
| --- | --- |
| The trial included individuals diagnosed with cancer.  The research topic involves the application and evaluation of mandala painting therapy's impact on cancer patients, encompassing aspects such as mandala painting therapy, mandala painting, and mandala therapy.  The literature review includes primary research such as randomized controlled trials, non-randomized controlled trials, case-control studies, cohort studies, case studies, qualitative studies, and so on.  The language used is either Chinese or English. | Literature on Mandala Painting Therapy, when combined with other interventions, demonstrates the effects of Mandala Painting. However, there is a lack of effective measurement for therapy outcomes.  The literature available is incomplete or ambiguous, with full texts often being unavailable.  Studies found in published literature have been replicated.  Non-peer-reviewed conference papers and posters are also included. |

Table 2 Characteristics of included studies and their frequencies.

|  | n (%) | Included studies |
| --- | --- | --- |
| Publication year |  |  |
| 2000-2010 | 9% | Elkis-Abuhoff D et al.(2009) |
| 2011-2022 | 73% | Yaoli Li et al.(2017),Shufen Zhao et al(2017),Shufen Zhao et al.(2019),Gürcan M and Atay Turan S(2020),Gürcan M and Atay Turan S(2021),Yakar H K et al.(2021),Lili Sun et al.(2021),Lingyu Su et al.(2022) |
| 2023 | 18% | Moharamkhani M et al.(2023),Akbulak F and Can G(2023) |
| Research design |  |  |
| Qualitative studies | 18% | Elkis-Abuhoff D et al.(2009),Gürcan M and Atay Turan S(2020) |
| Quantitative studies |  |  |
| Controlled trial | 55% | Yaoli Li et al.(2017),Shufen Zhao et al(2017),Shufen Zhao et al.(2019),Gürcan M and Atay Turan S(2020),Gürcan M and Atay Turan S(2021),Lili Sun et al.(2021),Lingyu Su et al.(2022) |
| Type of experimental studies | 27% | Yakar H K et al.(2021),Moharamkhani M et al.(2023),Akbulak F and Can G(2023) |
| Country of origin |  |  |
| China | 46% | Yaoli Li et al.(2017),Shufen Zhao et al(2017),Shufen Zhao et al.(2019),Lili Sun et al.(2021),Lingyu Su et al.(2022) |
| Turkey | 36% | Gürcan M and Atay Turan S(2020),Gürcan M and Atay Turan S(2021),Yakar H K et al.(2021),Akbulak F and Can G(2023) |
| United States Of America | 9% | Elkis-Abuhoff D et al.(2009) |
| Iran | 9% | Moharamkhani M et al.(2023) |

Table 3 General characteristics of the included studies

| Inclusion of literature | Published (year) | Country or region | Research object | Research design | Research objective | Interventionists | Time of intervention  /Duration | Forms of intervention |
| --- | --- | --- | --- | --- | --- | --- | --- | --- |
| Yakar H K et al.[15] | 2021 | Istanbul | Cancer patient | Type of experimental re- search | Period of inactive treatment | Trainers, researchers who have participated in training | One day per week for 8 weeks,2h/session | Unstructured mandalas accompanied by Far Eastern and classical music in languages other than the individual's native language. |
| Lili Sun et al.[16] | 2021 | China | Cancer patient | A ra- ndomised controlled trial | Period of chemotherapy | Teams that have participated in training,including counsellors, etc. | 2 cycles of chemotherapy per cycle for a total of 10 cycles | Structured mandala with calming music |
| LingYu Su et al.[17] | 2022 | China | Patients with primary liver cancer | A ra- ndomised controlled trial | Operative period | Teams that have participated in training,including counsellors,nurses,graduate stud- ents | 1 intervention per day for a total of 6 sessions of 50-60min duration | Structured mandala with soothing soft music |
| YaoLi Li et al.[18] | 2017 | China | Terminal cancer patient | A ra- ndomised controlled trial | End-stage cancer | nurses | Once a week, give enough time | Not mentioned, soothing soft music plus meditation |
| ShuFen Zhao et al.[19] | 2017 | China | Non-elderly cancer patients | A ra- ndomised controlled trial | Treatment period | Psychological counsellors, trained members of hospital psychological care teams, oncology nurses | 2 times a week, 45min each time, 15:00~15:45 | Structural Mandala, no |
| Gürcan M et al.[20] | 2021 | Istanbul | Adolescent cancer patients | A ra- ndomised controlled trial | End-stage cancer | Fellows who have attended mandala courses | Two times in total, at intervals of 2 or 3 days, with time taken into account for adolescents' recommendations | Unstructured mandala accompanied by classical or instrumental music |
| ShuFen Zhao et al.[21] | 2019 | China | Patients with esophageal cancer | A ra- ndomised controlled trial | Radiation therapy period | Members of hospital care psychology teams, psycholog -ists who have participated in the training | 1 time/week, 6 weeks in total, 40min/time, 15:30~16:10 | Structural Mandala, no |
| Akbulak F et al.[22] | 2023 | Istanbul | Female breast cancer patients | Type of experimental research | Period of chemotherapy | Nurses | Total 1 time, 30min | Structural Mandala, no |
| Moharamkhani M et al.[23] | 2023 | Iran | Childhood cancer patients | Type of experimental research | Not in advanced stages | Researcher | Daily, in 6 sessions for 6 days, 45 min each session (11:00 a.m.) | Structural Mandala, no |
| Gürcan M et al.[24] | 2020 | Istanbul | Adolescent cancer patients | Qualitative research | Treatment period | Researcher | Total 1 session, 1-2 hours | Unstructured mandala, accom -panied by classical or instrumental music |
| Elkis-Abuhoff et al.[25] | 2009 | United States of America | Female breast cancer patients | Qualitative research | Treatment period | Doctors | Every time you go to the doctor. | Unstructured mandala, no |

Table 4 Outcome indicator of the included studies

| Inclusion of literature | Psychological indicators | Other indicators |
| --- | --- | --- |
| Yakar H K et al.[15] | Distress thermometer  State/Trait Anxiety Inventory STAI | / |
| Lili Sun et al.[16] | positive  And negative affect scale,PANAS | / |
| LingYu Su et al.[17] | self-rating anxiety scale,SAS self-rating depression scale,SDS | pittsburgh sleep quality index,PSQI  Mandala painting therapy satisfaction questionnaire |
| YaoLi Li et al.[18] | self-rating anxiety scale,SAS self-rating depression scale,SDS | Nursing satisfaction questionnaire  Quality of life rating form |
| ShuFen Zhao et al.[19] | self-rating anxiety scale,SAS | / |
| Gürcan M et al.[20] | The Hospital Anxiety and Depression Scale,HADS  The Memorial Symptom Assessment Scale,MSAS | / |
| ShuFen Zhao et al.[21] | self-rating depression scale,SDS | quality of life questionnaire,QLQ-C30 |
| Akbulak F et al.[22] | The distress thermometer  The State–Trait Anxiety Inventory | / |
| Moharamkhani M et al.[23] | Spielberger State-Trait Anxiety Inventory | / |
| Gürcan M et al.[24] | / | / |
| Elkis-Abuhoff et al.[25] | / | / |


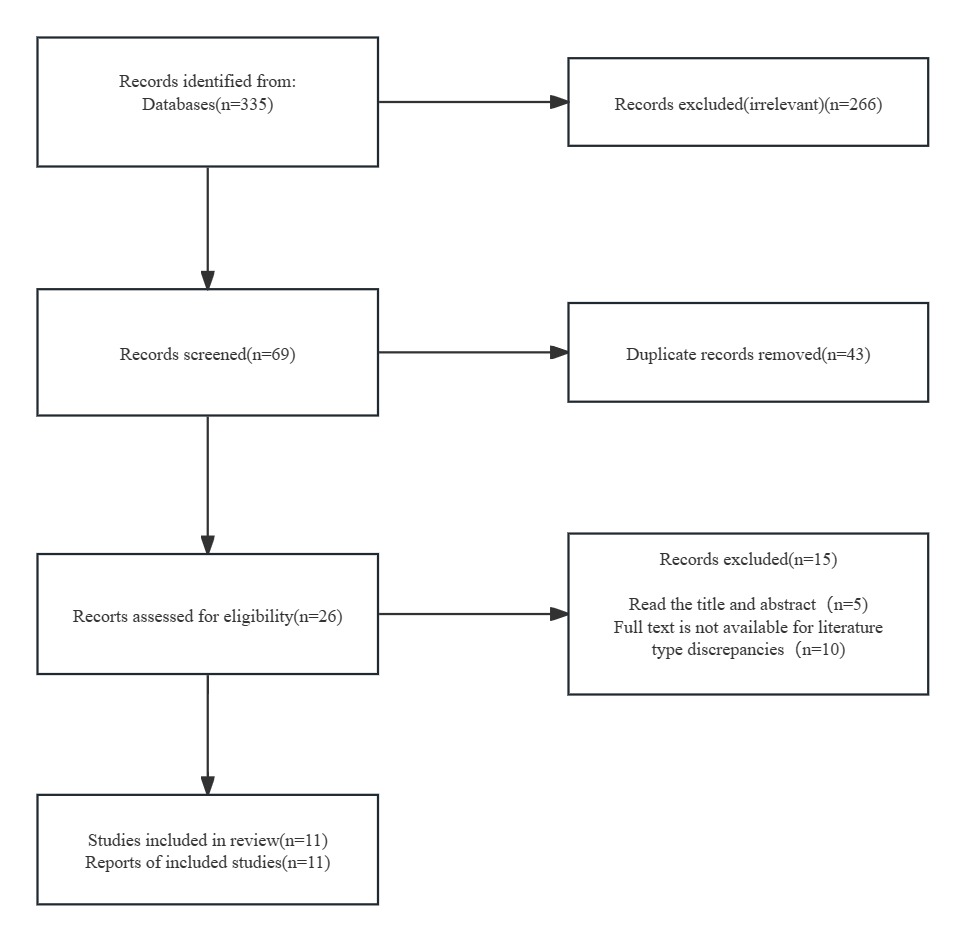


**Figure 1 Flow chart of literature screening**
